# Does Spinal Fusion and Scoliosis Correction Improve Activity and Participation for Children With GMFCS level 4 and 5 Cerebral Palsy?

**DOI:** 10.1097/MD.0000000000001907

**Published:** 2015-12-11

**Authors:** Mathew David Sewell, Charlie Wallace, Francesc Malagelada, Alex Gibson, Hilali Noordeen, Stewart Tucker, Sean Molloy, Jan Lehovsky

**Affiliations:** From the Spinal Deformity Unit, Royal National Orthopaedic Hospital, Stanmore, UK

## Abstract

Spinal fusion is used to treat scoliosis in children with cerebral palsy (CP). Following intervention, the WHO considers activity and participation should be assessed to guide intervention and assess the effects. This study assesses whether spinal fusion for scoliosis improves activity and participation for children with severe CP.

Retrospective cohort study of 70 children (39M:31F) with GMFCS level 4/5 CP and significant scoliosis. Thirty-six underwent observational and/or brace treatment as the sole treatment for their scoliosis, and 34 underwent surgery. Children in the operative group were older and had worse scoliosis than those in the observational group. Questionnaire and radiographic data were recorded over a 2-year period. The ASKp was used to measure activity and participation.

In the observational group, Cobb angle and pelvic obliquity increased from 51^o^ (40–90) and 10^o^ (0–30) to 70^o^ (43–111) and 14^o^ (0–37). Mean ASKp decreased from 16.3 (1–38) to 14.2 (1–36). In the operative group, Cobb angle and pelvic obliquity decreased from 81^o^ (50–131) and 14^o^ (1–35) to 38^o^ (10–76) and 9^o^ (0–24). Mean ASKp increased from 10.5 (0–29) to 15.9 (3–38). Spinal-related pain correlated most with change in activity and participation in both groups. There was no difference in mobility, GMFCS level, feeding or communication in either group before and after treatment.

In children with significant scoliosis and CP classified within GMFCS levels 4 and 5, spinal fusion was associated with an improvement in activity and participation, whereas nonoperative treatment was associated with a small reduction. Pain should be carefully assessed to guide intervention.

## INTRODUCTION

Cerebral palsy (CP) describes a group of permanent disorders of the development of movement and posture, causing activity limitation, that are attributed to nonprogressive disturbances that occurred in the developing fetal or infant brain. The motor disorders of CP are often accompanied by secondary musculoskeletal problems.^1^ Worldwide CP is the commonest cause of motor disability in childhood with an incidence 2 to 3 per 1000 live births^2^ and presents a wide spectrum of severity. The Gross Motor Function Classification System (GMFCS) groups children into 1 of 5 levels based on their ability to mobilize and reflects overall gross motor skills and severity of motor impairment.^3^ There is a linear relationship between the scoliosis risk and the GMFCS level; children within GMFCS levels 1 to 2 have very low risk, 3 intermediate, and 4 to 5, very high risk.^4^ Scoliosis occurs in 50% of children with GMFCS levels 4 or 5 CP.^4^ The scoliotic curve is also more severe in these children, and more likely to progress. Severe scoliosis produces pelvic obliquity affecting the sitting ability of children. Secondarily this may cause pain, pressure sores, and difficulty with hygiene. Many children undergo spinal fusion to correct the deformity and prevent progression. This is a major, life-threatening intervention; therefore, there is an imperative to evaluate this intervention using outcomes that are more meaningful to patients and their carers.^5^

The World Health Organization's (WHO) International Classification of Functioning, Disability, and Health (ICF) has redefined the way disability is viewed for children with impairments.^6^ There is less focus on actual impairments, and more on their impact for involvement in life situations, which is defined as “participation.” In the absence of a cure, optimizing quality of life (QoL) and activity and participation are now considered some of the more important outcome measures for children with complex neurodisability.^5–9^ Children with CP have lower participation than children in the general population, and those with more impairments have lower participation than those with fewer impairments. Participation differs from QoL, which is less influenced by the type and severity of impairments, and is similar between children with CP and the general population.^7^ The WHO recommend both should be assessed for children with CP to guide intervention and assess the effects.^8,9^

The aim of spinal fusion for children with GMFCS level 4/5 CP is to restore trunk alignment, prevent deterioration in respiratory function, alleviate pain caused by impingement of the ribs against the iliac crest on the concave side of the curve, improve personal hygiene, and provide better sitting tolerance in the wheelchair. Systematic review data have shown minimal evidence exists to advise parents about QoL changes,^10^ and there are no reports on the effects of spinal fusion on activity and participation. Furthermore most studies group all children with CP into the same category and do not differentiate between GMFCS levels, which fails to acknowledge the wide spectrum of severity. In this study we report outcome of scoliosis treatment for children with severe forms of CP (classified within GMFCS levels 4/5), with particular focus on activity and participation, and investigate factors that affect this.

## METHODS

### Participants

Between 2007 and 2012, a retrospective cohort study including children with GMFCS level 4 or 5 CP and scoliosis was performed. The diagnosis of CP was based upon clinical features and magnetic resonance imaging.^11^ Children were included if they had GMFCS level 4 or 5 CP, were in the age group between 8 and 17, and had a Cobb angle >40^o^,^12^ as this was the curve severity and age that the treating physicians would consider some form of intervention in childhood. The cohort comprised 70 children (39 males and 31 females) with a mean age of 12.9 years (8–17).

**FIGURE 1:**
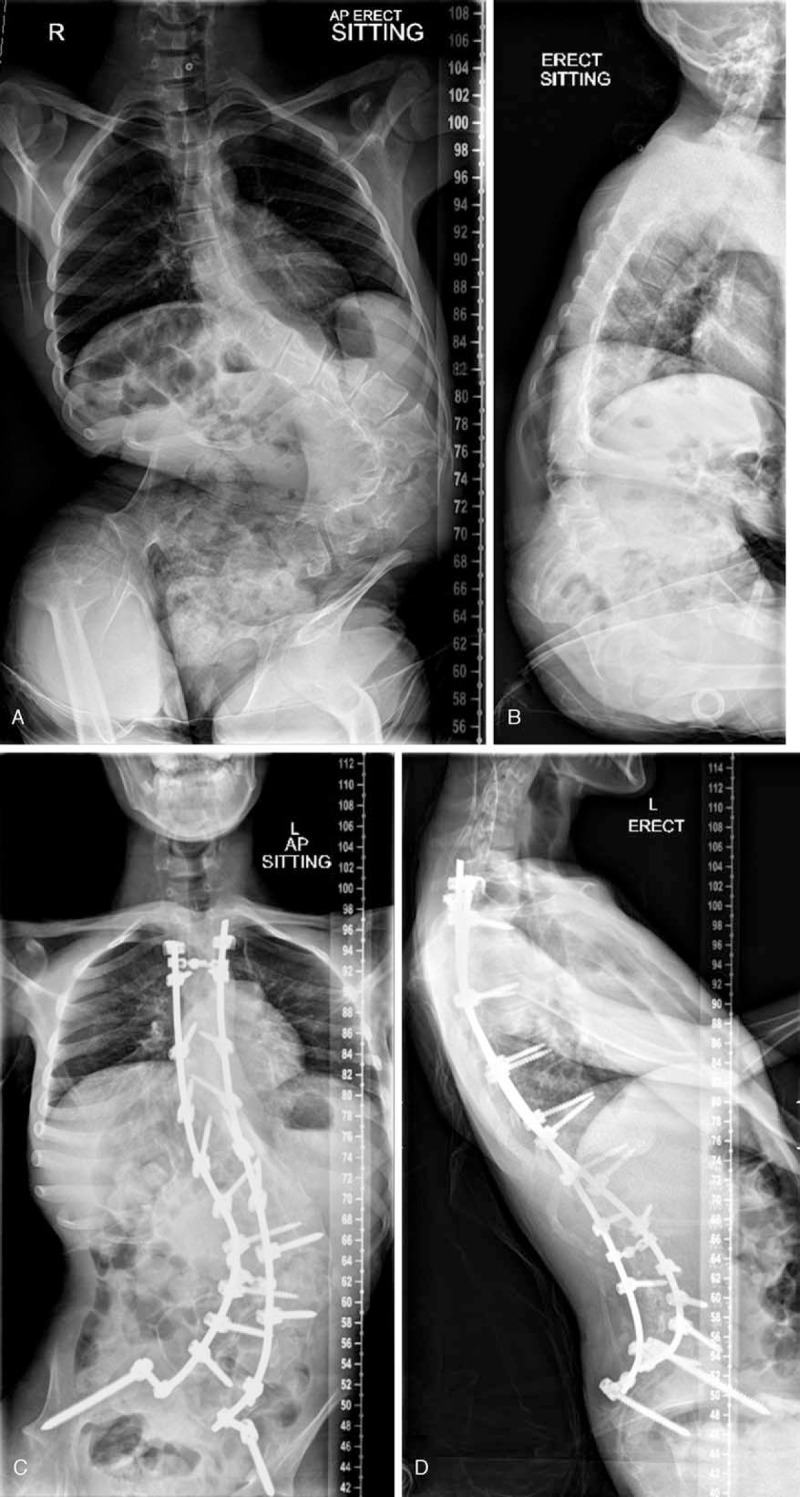
Preoperative antero-posterior (A) and lateral (B) radiographs of a 14-year old boy with GMFCS level 4 CP. The child was having difficulty sitting upright and repeated chest infections. Postoperative antero-posterior (C) and lateral (D) radiographs at 2 years following 2-stage posterior spinal fusion with segmental pedicle screw fixation from T2 to pelvis. The child had better sitting posture and suffered fewer chest infections. CP = cerebral palsy, GMFCS = gross motor function classification system.

Thirty-six underwent seating adaptations and/or brace treatment as the sole treatment for their scoliosis during the study period, and 34 underwent spinal fusion surgery. The indication for surgery was a coronal deviation of the spine >40^o^, with evidence of progression on sequential radiographs, in a child in whom the parent and surgeon both thought this curvature was contributing to pain or impaired function, hygiene, or social interaction (Fig. 1). Observational management with seating adaptations and/or brace treatment was indicated for all children with a scoliosis >40^o^, who did not meet these criteria, or were considered unfit for surgery due to anesthetic reasons. In the surgical group, 6 children underwent a 2-stage procedure (anterior release followed 1 week later by an instrumented posterior spinal fusion), and 28 underwent a single stage instrumented posterior spinal fusion. The mean number of fused levels was 14.8 (8–16). Twenty-one had instrumented fixation to the pelvis and 14 did not. Pelvis fixation was indicated when pelvic obliquity was thought to be contributing to worse sitting function in the wheelchair. Demographics and impairment characteristics of children in each group are shown in Table 1. Data from questionnaires administered to caregivers and children, clinic reviews, medical records, and radiographs were used for analysis over a 2-year period. This was a retrospective, observational study and local ethical review classified the study as a service evaluation.

**TABLE 1:**
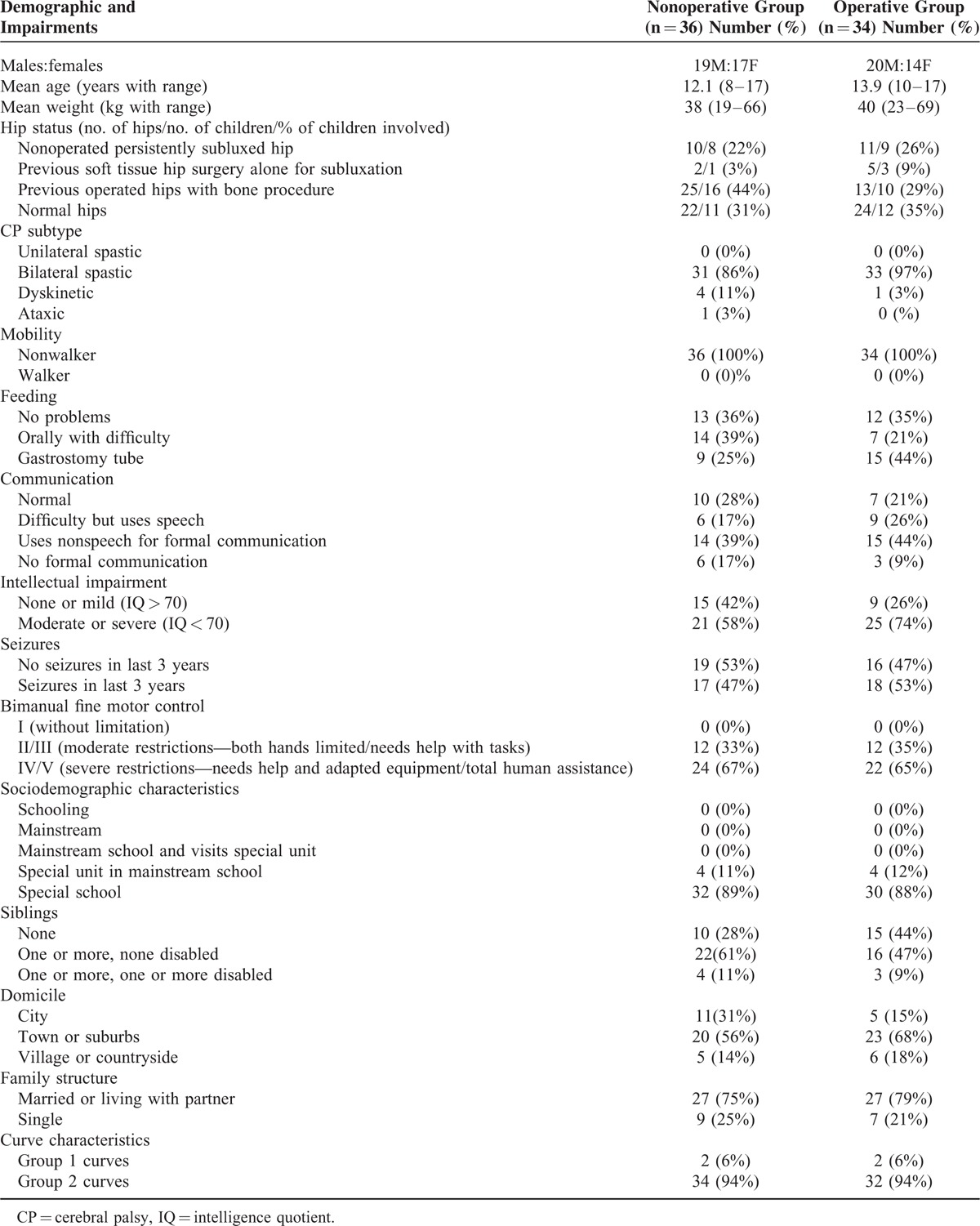
Demographics and Impairment Characteristics of Children in Both Groups

## MEASUREMENTS

### Activity and Participation Measurement Questionnaire

The performance version of the Activities Scale for Kids (ASKp) questionnaire was used to assess activity and participation.^13^ The ASKp comprises 30 items on personal care (3 items), dressing (4 items), locomotion (7 items), standing skills (5 items), other skills (4 items), transfers (5 items), and play (2 items) that are aggregated into 1 overall summary score. The ASKp is a self-report measure designed for children 5 to 15 years old, experiencing limitations in physical activity due to musculoskeletal disorders. The child (or the parent if the child is unable to do so) indicates how often each activity was performed in the last week. The 5 ordinal responses to each item are: “all of the time,” “most of the time,” “sometimes,” “once in a while,” and “none of the time.” The summary score is the average of all completed items multiplied by 25 in order to convert to a score ranging from 0 to 100, where high scores are indicative of greater activity and participation.

The ASKp has been widely used to assess activity and participation in children with CP, and multiple independent reviews have consistently reported the ASKp to have excellent psychometric properties and reliability, and be one of the optimal measures of activity and participation for these children.^14,15^ Parents were asked if their child would be able to understand the questionnaire for self-report. In those children considered not capable of self-report, the parents completed the questionnaire on behalf of their child, either in clinic or while at home. Parent and child-completed questionnaires provide very similar scores.^13^ In children who underwent surgery, the ASKp questionnaire was completed preoperatively and at 2 years. In children who did not undergo surgery, the ASKp was completed when the scoliosis was >40^o^, and 2 years thereafter.

### Gross Motor Function and Associated Impairments

Parents provided information about their area of domicile, and about their child's seizures, feeding, communication, intellect, school type, siblings, and gross motor function. Sociodemographic and impairment characteristics were assessed as these can affect activity and participation outcomes. The gross motor function level was classified according to the GMFCS,^3^ which is a reliable and valid instrument.^16^ The age-dependant GMFCS groups children into 1 of 5 levels based on their ability to mobilize and reflects overall gross motor skills and severity of motor impairment. Level 1 (walks and climbs stairs, without limitation) represents the highest level of gross motor function and level 5 (unable to walk, severely limited self-mobility) the lowest. Pain was assessed on a 5-point subjective scale: none, very mild, mild, moderate, or severe. This was adapted from the pain severity question on the Child Health Questionnaire^17^ and previous studies assessing associations between the pain and the health status in children with CP.^8^

### Radiographic Measurements

Radiographic data including Cobb angle (measure of coronal plane scoliosis deformity),^12^ pelvic obliquity (marker of pelvic position in the coronal plane which is relevant for sitting function), lumbar lordosis between L1 and L5 and thoracic kyphosis between T2 and T12 (measures of sagittal plane deformity) were also recorded. Scoliosis was classified as idiopathic-like (Group 1) or collapsing neuromuscular type (Group 2), according to the criteria of Lonstein and Akbarnia.^18^

### Statistical Analysis

The statistical program SPSS version 19 (IBM, US) was used. Pre and post-treatment ASKp scores within the same group were compared using the paired *t* test for matched data. Pre and post-treatment ASKp scores between groups were compared using the unpaired *t* test for nonpaired data. Baseline differences between groups were compared using the chi squared test for ordinal data, and Mann–Whitney *U* for continuous. Pearson correlation was performed at a 2-tailed level to determine significant relationships between variables. We coded responses to ordinal and nonlinear variables (eg mode of communication) into binary outcomes (eg normal speech or difficulty but uses speech versus uses nonspeech for formal communication or no formal communication) for the correlation analysis. The sample was inadequately powered for multivariate analysis with controlling for confounding variables. A *P* value < 0.05 was considered significant.

## RESULTS

### Baseline Differences Between Groups

At the start of the study, children in the operative group were older (*P* < 0.05), had more spinal-related pain (*P* < 0.05), worse ASK scores (*P* = 0.003), and Cobb angles (*P* < 0.001) than those in the observational group. There was no statistical difference in the GMFCS level (*P* = 0.27), feeding (*P* = 0.15), communication (*P* = 0.51), seizures (*P* = 0.82), intellectual impairment (*P* = 0.18), CP sub-type (*P* = 0.25), or weight (*P* = 0.49).

### Activity and Participation

The ASKp improved in all children who underwent surgery (*P* < 0.01) (Table 2). This was mainly due to improved sitting balance and less pain following the surgery. In the nonoperative group, 28 of the 36 children reported a reduction in the ASKp at 2 years (*P* < 0.01).

**TABLE 2:**
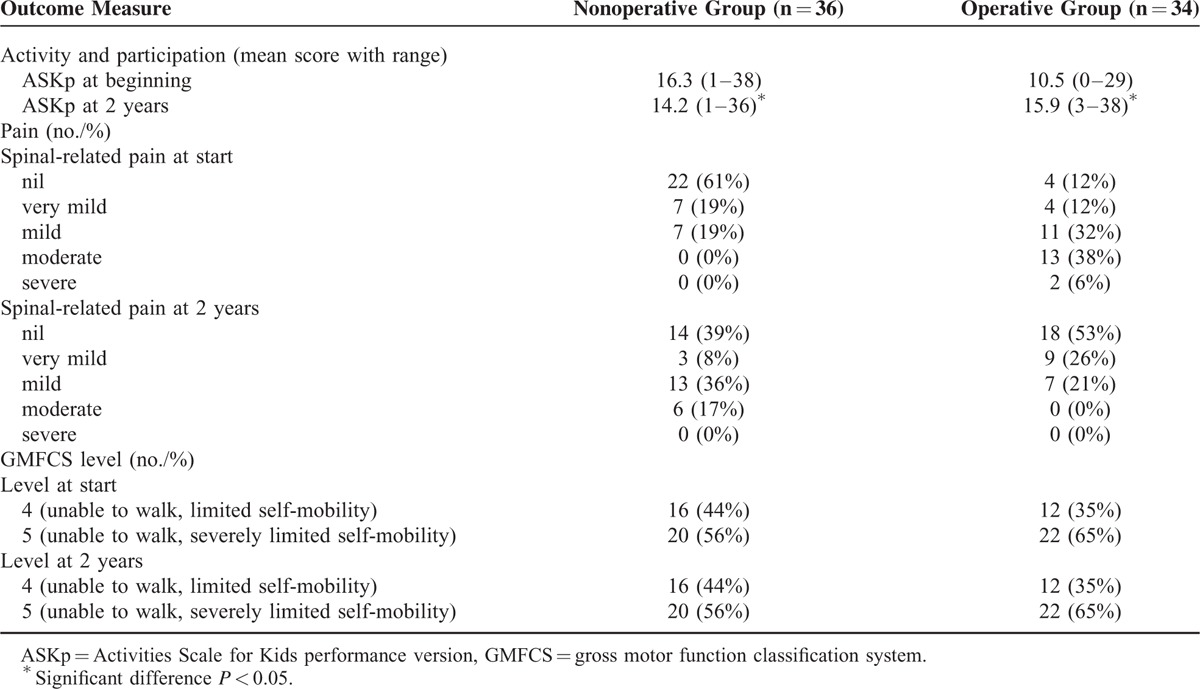
Activity and Participation, Pain, and Mobility Outcomes Over 2-Year Study Period

### Factors Affecting Activity and Participation

In the operative group, change in pain from moderate or severe to mild or none strongly correlated with a greater improvement between pre- and postoperative ASKp scores (*r* = 0.840, *P* < 0.001). There was no significant correlation between incidence of postoperative complications (*r* = −0.120, *P* = 0.50), fusion to the pelvis (*r* = 0.085, *P* = 0.63), postoperative Cobb angle (*r* = 0.19, *P* = 0.29), change in Cobb angle (*r* = 0.10, *P* = 0.67), thoracic kyphosis (*r* = 0.003, *P* = 0.99), pelvic obliquity (*r* = −0.02, *P* = 0.93), GMFCS level (*r* = −0.25, *P* = 0.16), mode of communication (*r* = 0.18, *P* = 0.32), or presence of persistent hip dislocation (*r* = 0.17, *P* = 0.33), and mean improvement in pre- and postoperative ASKp scores.

In the operative group, although the GMFCS level did not correlate with mean difference in pre- and postoperative ASK scores, the GMFCS level did correlate with ASK scores at the start of the study (*r* = −0.73, *P* < 0.001) and at 2 years (*r* = −0.75, *P* < 0.001). Children with GMFCS level 4 function had better ASKp scores than those with GMFCS level 5 function throughout the study (*P* < 0.05).

In the observational group, change in pain from none or mild to moderate or severe demonstrated a weak correlation with greater reduction between mean pre- and postoperative ASKp scores (*r* = −0.306, *P* = 0.069), without statistical significance. The GMFCS level did not correlate with mean difference in ASK scores (*r* = 0.13, *P* = 0.44); however GMFCS levels did correlate with ASK scores at the start of the study (*r* = −0.49, *P* = 0.003) and end (*r* = −0.41, *P* = 0.01). Children with GMFCS level 4 function had better ASKp scores than those with GMFCS level 5 function throughout the study (*P* < 0.05).

### Changes in Pain, Associated Impairments, and Radiographic Measurements

There was a reduction in the number of children experiencing pain in the operative group. There was an increase in the number of children experiencing pain in the nonoperative group. Pain was attributed to worse sitting balance in the nonoperative group. There was no difference in mobility, GMFCS level, feeding, or communication in either group before and after treatment. Changes in radiographic parameters are shown in Table 3.

**TABLE 3:**
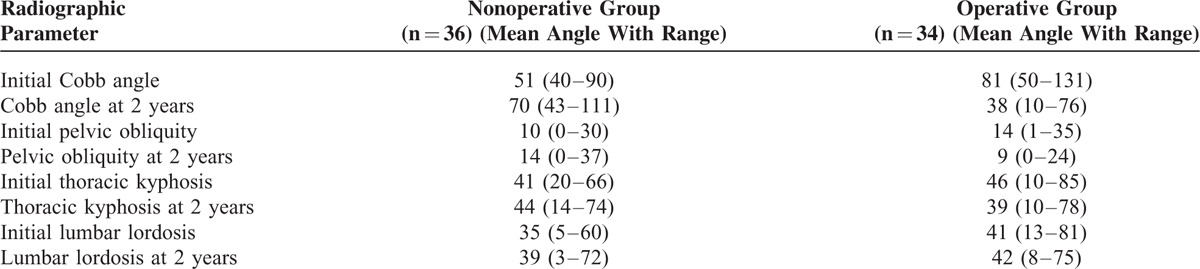
Radiographic Outcomes

### Complications in the Operative Group

Six children developed wound infections (18%) requiring intravenous antibiotics; 2 required additional surgical debridement for infection control. Five developed chest infections (15%) requiring intravenous antibiotics and respiratory support. Two developed pneumothoraces, which resolved. One child, who had previously undergone open reduction and proximal femoral osteotomy for a dislocated hip, developed recurrent dislocation at 1 year. The incidence of complications did not correlate with mean change in ASKp scores (*r* = −0.120, *P* = 0.50).

## DISCUSSION

Preliminary results from this study suggest that spinal fusion for significant scoliosis in children with CP classified within GMFCS levels 4 and 5 was associated with an improvement in activity and participation and decrease in spinal-related pain, whereas nonoperative treatment was associated with a small reduction in activity and participation and increase in pain. Spinal-related pain was the factor that correlated most with change in activity and participation in both groups. Surgery was not associated with a change in mobility, GMFCS level, feeding, or communication. Persistent hip dislocation or pelvic fusion did not correlate with improvement in activity and participation in the surgical group.

This was an observational study tracking a younger group of children with CP and significant scoliosis as their scoliosis worsened during childhood, and an older group with more severe scoliosis who underwent spinal fusion. Groups were not comparable even though relatively strict inclusion criteria were used. Children in the operative group had worse Cobb angles were more likely to have spinal-related pain and were a mean 1.8 years older than children in the nonoperative group. They also had worse ASKp scores at the start of the study. It is likely that these systematic differences between groups explain why the most significant changes were observed in these variables (Cobb angle, pain, ASKp score) following intervention. Children in the observational group, despite deteriorating in these parameters over the course of the study, did not deteriorate to the same levels as observed in the operative group. All included children in the study had the magnitude of scoliosis to indicate surgery; however the indication for surgery was a joint decision between surgeon and caregiver and the views of the caregiver were the most important when deciding to proceed with surgery. This was primarily based on the presence of spinal-related pain or impaired function, hygiene, or social interaction. We did not identify any significant difference between groups with respect to baseline impairments, although it is important to note that children in the operative group had worse feeding ability than those in the nonoperative group. We did not analyze how weight changed during the treatment period, but this could be relevant to account for change in ASKp scores.

There are no studies reporting changes in activity and participation following scoliosis treatment for children with CP. The optimal way to measure activity and participation in children with complex disability is still being debated, and no single measure adequately addresses all aspects of the WHO ICF.^6,14,15,19–22^ In children with CP, systematic review evidence has shown that the ASKp has the most robust psychometric properties for activity, with other tools requiring further confirmation of validity (measures what it intends to do), reliability (consistent and free from error), and responsiveness.^14^ However, the ASKp still requires further examination of responsiveness,^14^ and in the author's opinion, particularly its application and responsiveness in the more severely impaired children (GMFCS 5). Alternative measures that may be considered for future studies include the Children's Assessment of Participation and Enjoyment (CAPE),^20^ the lifestyle assessment questionnaire (LAQ-CP),^21^ and the participation and environment measure for children and youth (PEM-CY).^22^

Children in our study had significant gross motor function impairment with low baseline ASKp scores, worse than would be present in a representative population of children with GMFCS level 4/5 CP without scoliosis.^23^ This may have undervalued the true benefit surgery conferred these children, as we only found a small improvement in the mean ASKp following surgery (5.4 points), although this represents a 50% improvement when compared to the mean baseline preoperative score. When compared to historical controls,^23^ children with scoliosis and GMFCS level 4/5 CP in this study had lower activity and participation scores than those with GMFCS level 4/5 CP and no scoliosis.

In the operative group, change in pain from moderate or severe to mild or none strongly correlated with greater improvement in ASKp scores. In the observational group, development of moderate or severe pain weakly correlated with a greater reduction in ASKp scores. This latter statistic did not reach significance, which likely represents type 2 error. Pain is therefore an important factor that affects activity and participation in children with CP and scoliosis and should be carefully assessed. This finding is similar to previous multicentre cross-sectional studies that have shown pain is a common experience affecting QoL and participation for all children with CP.^7,8,24^ The clinical implications being that clinicians should ask about pain when they see a child with CP and scoliosis, as children with CP might always have lived with pain and assume it to be normal. Clinicians may start with simple interventions such as analgesia given before physiotherapy and attentive orthotic management; however if these measures fail, we have shown surgery for children with severe scoliosis may provide an alternative option to decrease pain and improve activity and participation.

Persistent hip dislocation did not correlate with change in activity and participation in the surgical group. Therefore when both hip dislocation and scoliosis exist simultaneously, it would seem reasonable to target intervention at the most painful pathology first. We chose a 2-year follow-up time to assess outcome following initiation of treatment, as previous literature suggests there is a recovery period after spinal surgery for neuromuscular patients associated with an initial decrease in functional skills, with improvements usually apparent by 12 to 24 months.^25^

### Study Limitations

This study has significant limitations due to methodological challenges and design. Baseline characteristics for children in the operative and observational groups differed in several domains. This will have introduced bias. Children were 1.8 years older, had worse Cobb angles and more spinal-related pain in the surgical group. These factors will have influenced the decision to manage nonoperatively or operatively and affected ASKp scores. The sample was inadequately powered for multivariate analysis with controlling for confounding variables, which may have introduced type 2 error, although no formal power analysis was done. Different researchers obtained ASKp scores, which might have introduced systematic differences into parent and child responses. The ASKp questionnaire is frequently used to assess activity and participation in children with CP; however it may lack validity, sensitivity, and responsiveness in the more severely impaired children and underestimate the true value of the procedure. A randomized trial is not ethical or practical and previous studies that have assessed QoL in children with CP undergoing scoliosis surgery have lacked comparison groups to enable comparison.^10^ Given the paucity of published information and methodological challenges associated with assessing activity and participation in these children,^26,27^ this study provides preliminary results for clinicians to advise families of children with this condition and plan future research. Recommendations for future prospective studies include using additional measures sensitive to activity for this population (eg frequency of repositioning, sitting tolerance time, sitting balance, sitting pressures, pressure ulcers, respiratory function, constipation, and reflux) and using a more robust pain measurement instrument.

## CONCLUSIONS

In children with significant scoliosis and CP classified within GMFCS levels 4 and 5, spinal fusion was associated with an improvement in activity and participation, whereas nonoperative treatment was associated with a small reduction. Spinal-related pain was the factor that correlated most with change in activity and participation in both groups and therefore should be carefully assessed to help guide intervention. These results should be interpreted with caution due to methodological challenges with study design.
